# Nitrifier Gene Abundance and Diversity in Sediments Impacted by Acid Mine Drainage

**DOI:** 10.3389/fmicb.2017.02136

**Published:** 2017-11-07

**Authors:** Bhargavi Ramanathan, Andrew M. Boddicker, Timberley M. Roane, Annika C. Mosier

**Affiliations:** Department of Integrative Biology, University of Colorado Denver, Denver, CO, United States

**Keywords:** acid mine drainage, nitrification, ammonia-oxidizing archaea, ammonia-oxidizing bacteria, nitrite-oxidizing bacteria

## Abstract

Extremely acidic and metal-rich acid mine drainage (AMD) waters can have severe toxicological effects on aquatic ecosystems. AMD has been shown to completely halt nitrification, which plays an important role in transferring nitrogen to higher organisms and in mitigating nitrogen pollution. We evaluated the gene abundance and diversity of nitrifying microbes in AMD-impacted sediments: ammonia-oxidizing archaea (AOA), ammonia-oxidizing bacteria (AOB), and nitrite-oxidizing bacteria (NOB). Samples were collected from the Iron Springs Mining District (Ophir, CO, United States) during early and late summer in 2013 and 2014. Many of the sites were characterized by low pH (<5) and high metal concentrations. Sequence analyses revealed AOA genes related to *Nitrososphaera*, *Nitrosotalea*, and *Nitrosoarchaeum*; AOB genes related to *Nitrosomonas* and *Nitrosospira*; and NOB genes related to *Nitrospira*. The overall abundance of AOA, AOB and NOB was examined using quantitative PCR (qPCR) amplification of the *amoA* and *nxrB* functional genes and 16S rRNA genes. Gene copy numbers ranged from 3.2 × 10^4^ – 4.9 × 10^7^ archaeal *amoA* copies ∗ μg DNA^-1^, 1.5 × 10^3^ – 5.3 × 10^5^ AOB 16S rRNA copies ∗ μg DNA^-1^, and 1.3 × 10^6^ – 7.7 × 10^7^ *Nitrospira nxrB* copies ∗ μg DNA^-1^. Overall, *Nitrospira nxrB* genes were found to be more abundant than AOB 16S rRNA and archaeal *amoA* genes in most of the sample sites across 2013 and 2014. AOB 16S rRNA and *Nitrospira nxrB* genes were quantified in sediments with pH as low as 3.2, and AOA *amoA* genes were quantified in sediments as low as 3.5. Though pH varied across all sites (pH 3.2–8.3), pH was not strongly correlated to the overall community structure or relative abundance of individual OTUs for any gene (based on CCA and Spearman correlations). pH was positivity correlated to the total abundance (qPCR) of AOB 16S rRNA genes, but not for any other genes. Metals were not correlated to the overall nitrifier community composition or abundance, but were correlated to the relative abundances of several individual OTUs. These findings extend our understanding of the distribution of nitrifying microbes in AMD-impacted systems and provide a platform for further research.

## Introduction

Nitrification, a central part of the nitrogen cycle, is globally important because it transfers nitrogen to higher organisms and mitigates nitrogen pollution when coupled with denitrification and anammox. Nitrification is the two-step aerobic oxidation of ammonia to nitrate through nitrite. Ammonia oxidation is mediated by ammonia-oxidizing archaea and bacteria (AOA and AOB), while nitrite oxidation is mediated by nitrite-oxidizing bacteria (NOB) commonly from the *Nitrobacter* and *Nitrospira* lineages. Recent discoveries documented the complete oxidation of ammonia to nitrate (comammox) by *Nitrospira* (Daims et al., 2015; van Kessel et al., 2015). The microbial communities involved in ammonia oxidation and nitrite oxidation are commonly evaluated using 16S rRNA genes (Stephen et al., 1998) or functional markers such as the *amoA* gene (encoding the alpha subunit of the ammonia monooxygenase enzyme) (Stephen et al., 1999; Francis et al., 2005) and *nxrB* gene (encoding the beta subunit of the nitrite oxidoreductase enzyme) (Pester et al., 2014). Many nitrifiers are obligate ammonia oxidizers or nitrite oxidizers (based on genomic sequences or physiology in culture), though some have mixotrophic capabilities (Ward et al., 2011; Hatzenpichler, 2012).

Nitrification has been documented in virtually every environment across the earth (e.g., soil, marine, freshwater) (Ward et al., 2011). Nonetheless, very little is known about nitrification in systems impacted by acid mine drainage (AMD), which refers to acidic and metal-rich waters that flow out of coal and metal mines. One study found that nitrification was completely halted in some AMD-impacted streams when pH dropped below 5.3 (Niyogi et al., 2003). However, it is unknown whether the findings are a general phenomenon in other streams or whether AMD differentially impacts the various groups of microorganisms involved in nitrification.

Though the impacts of AMD on nitrification are largely unknown, other research has evaluated individual environmental factors that are often associated with AMD, including acidic pH and high metal concentrations. Nitrification is impacted by acidic pH, in part due to the reduced bioavailability of ammonia and nitrite at low pH (Suzuki et al., 1974; Stein et al., 1997). In some aquatic environments, overall nitrification rates were inhibited at pH values lower than 5.7–6 (Rudd et al., 1988; Huesemann et al., 2002). Small decreases in pH (by 0.05–0.14) reduced ammonia oxidation rates in the Atlantic and Pacific Oceans (Beman et al., 2011). In wastewater batch reactors, nitrite oxidation rates ceased at pH values lower than 6.5 (Jiménez et al., 2011). Nitrite oxidation rates by neutrophilic *Nitrobacter* and *Nitrospira* cultures significantly decreased below pH 6.5 (Ehrich et al., 1995; Grunditz and Dalhammar, 2001; Blackburne et al., 2007).

While acidic pH impacts the overall rates of ammonia oxidation and nitrite oxidation, some nitrifying microbes are capable of withstanding low pH conditions. AOA have been shown to be both abundant and active in some acidic soils, and often numerically dominate the AOB (e.g., Nicol et al., 2008; Gubry-Rangin et al., 2010, 2011; He et al., 2012; Zhang et al., 2012). Both AOA and AOB genes have been found in soils with pH values as low as 3.8 (He et al., 2007; Hu et al., 2013; Lu and Jia, 2013). AOA *amoA* genes and transcripts have been detected in acidic fen soil pore water with pH values ranging from 4.6–4.9 (Herrmann et al., 2012). A small number of 16S rRNA gene sequences related to AOA were described in an acid pit lake and in AMD sediments with pH 2–3.5 (Volant et al., 2012; Lucheta et al., 2013). Two acidophilic AOA cultured from soils showed growth at pH ranging from 4.0-6.1 (Lehtovirta-Morley et al., 2011, 2014).

High concentrations of metals in AMD-impacted systems may also affect nitrification, as has been shown in other environments. Heavy metals, such as copper, zinc, lead, cadmium, nickel, and metal sulfides, are associated with decreased nitrification rates in soils and freshwater sediments (Wilson, 1977; Broberg, 1984; Smolders et al., 2001; Cela and Sumner, 2002). Studies have demonstrated that AOA and AOB respond differently to heavy metals: AOA seemed more tolerant to copper and arsenic contamination than AOB in soils (Li et al., 2009; Subrahmanyam et al., 2014); however, AOB were more active than AOA in zinc contaminated soils (Mertens et al., 2009). The mechanism of metal resistance in ammonia oxidizers and nitrite oxidizers is largely unknown; however, metal resistance genes (e.g., copper, mercury, arsenic, zinc resistance) have been found in some nitrifiers, including *Nitrosomonas eutropha*, *Nitrososphaera gargensis*, *Nitrospira defluvii*, and *Nitrobacter hamburgensis* (Stein et al., 2007; Starkenburg et al., 2008; Lücker et al., 2010; Spang et al., 2012).

In the Colorado Rocky Mountains, AMD is a particularly common threat due to the large number of mines in the area. In the present study, we used high-throughput sequencing and quantitative PCR (qPCR) to examine the abundance and diversity of AOA, AOB, and NOB in AMD-impacted sediments in the Iron Springs Mining District near Ophir, CO, United States.

## Materials and Methods

### Site Description and Sample Collection

**FIGURE 1:**
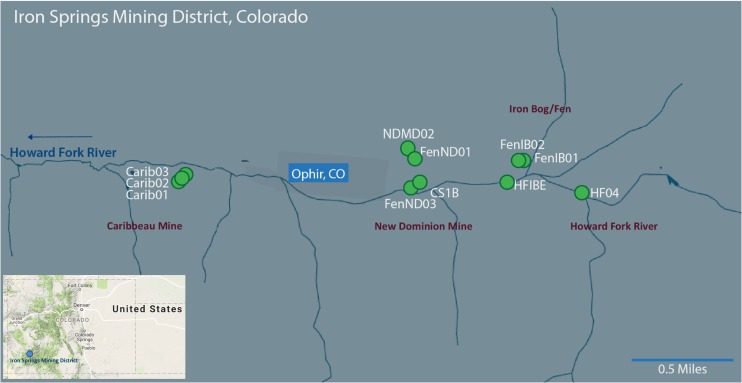
Map of the study sites within the Iron Springs Mining District near Ophir, Colorado. Individual sample sites are denoted with a green circle. Sampling regions are denoted in purple. Arrow indicates the direction of flow for the Howard Fork River. Base images modified from Google Maps (Map data Google 2015 and © Google 2017).

The Iron Springs Mining District located in Ophir, CO, United States consists of several abandoned mines. From 1877 to 1960, the Iron Springs Mining District was predominantly mined for metals such as silver, gold and lead, and also to some extent for iron (pigment) and tungsten (Nash, 2002). AMD from these mines drains directly into the Howard Fork River. Composite sediment samples (shoveled from the surface to a depth of approximately five centimeters) were collected from 11 sampling sites during June and August 2013 (Sackett and Roane, Master’s Thesis, University of Colorado, Denver, CO, United States), and from 10 sampling sites during June and September 2014 at Iron Springs (**Figure 1** and Supplementary Figure S1). Sediment samples for DNA extracts were stored on dry ice until permanent storage at -20°C in the laboratory freezer. For total recoverable metal (TRW) analysis, 500 mL of surface water sample was collected, acidified to pH <2 with concentrated nitric acid and stored at 4°C. For dissolved metal (DM) analysis, 500 mL of surface water sample was collected, filtered with a cellulose nitrite membrane filter (Thermo Scientific, Waltham, MA), acidified to pH <2 with concentrated nitric acid and stored at 4°C.

### Environmental Parameters

Temperature, pH, conductivity, and dissolved oxygen were measured at the sediment surface using a Thermo Scientific Orion 5-Star Multiparameter Meter Kit (Thermo Fisher Scientific, Inc., Waltham, MA, United States) and an *In Situ* Multiparameter meter. Metal analyses (TRW and DM) of the Iron Springs water samples were done at the EPA Region 8 lab (Golden, CO, United States) using Inductively-Coupled Plasma Mass Spectrometry (ICP-MS) following EPA method 200.8, and Inductively-Coupled Plasma Optical Emission Spectrometry (ICP-OES) following EPA method 200.7. The analytes measured for TRW and DM included aluminum (Al), antimony (Sb), arsenic (As), barium (Ba), cadmium (Cd), calcium (Ca), copper (Cu), iron (Fe), lead (Pb), magnesium (Mg), manganese (Mn), nickel (Ni), selenium (Se), silver (Ag), sodium (Na), strontium (Sr), thallium (Tl), vanadium (V) and zinc (Zn).

### DNA Extraction

The PowerMax Soil DNA Isolation Kit (MO BIO Laboratories, Inc., Carlsbad, CA, United States) was used to isolate total DNA from subsamples of mechanically homogenized saturated composite sediment (∼10 g) from each sample site. DNA extracts were quantified using the Qubit dsDNA HS Assay Kit with the Qubit 2.0 Fluorometer (Life Technologies Corporation, Carlsbad, CA, United States). High quality samples (*n* = 38) based on gel electrophoresis, DNA quantification, and PCR amplification were selected for further analyses (excluded samples were HF04-June14; HF04-Sept14; FenND03-Jun13; FenND03-Sept14; FenIB02-Sept14; Carib01-June14).

### Quantification of Gene Abundance

AOA, AOB, and NOB genes were quantified using the StepOnePlus Real–Time PCR system (Life Technologies Corporation). AOA *amoA* genes were quantified using the primers Arch–amoAF and Arch-amoAR (Francis et al., 2005). AOB were quantified using AOB-specific 16S rRNA gene primers NitA and CTO654r (Voytek and Ward, 1995; Kowalchuk et al., 1997; Sonthiphand et al., 2013). AOB *amoA* genes did not amplify in our samples using primers amoA-1F/amoA–2R (Rotthauwe et al., 1997) or amoA–1F∗/amoA–2R (Stephen et al., 1999). *Nitrospira nxrB* genes were quantified using the primers nxrB169F and nxrB638R (Pester et al., 2014). Three primer sets were used in an attempt to amplify *Nitrobacter* genes: *nxrB* gene primers nxrB-1F/nxrB-1R (Vanparys et al., 2007), *Nitrobacter*–specific 16S rRNA gene primers Nitro-1198f/Nitro-1423r (Graham et al., 2007; Huang et al., 2010), and *Nitrobacter*–specific 16S rRNA gene primers FGPS872f/FGPS1269r (Degrange and Bardin, 1995).

qPCR conditions and reactions (with modifications pertaining to the current study) were carried out as previously described for archaeal *amoA* (Mosier and Francis, 2008), and for AOB 16S rRNA (Sonthiphand et al., 2013) with the addition of a plate read step at 81°C for 10 s at the end of each cycle. The previously described PCR conditions for *Nitrospira nxrB* (Pester et al., 2014) were applied to qPCR with the addition of a plate read step at 84°C for 10 s at the end of each cycle. The reactions were performed in a 20 μl reaction mixture with 1 μl of template DNA, 0.4 μl of 25 μM passive reference dye, and the following for the respective primer pairs: 10 μl of Failsafe Green Premix E (Epicentre, Madison, WI, United States), 2 mM of MgCl_2_, 0.4 μM of each primer, 40 ng ∗ μl^-1^ of BSA, and 1 Unit of AmpliTaq DNA Polymerase (Applied Biosystems, Foster City, CA, United States) for archaeal *amoA*; 10 μl of Failsafe Green Premix F (Epicentre, Madison, WI, United States), 0.3 μM of each primer, 19.8 ng ∗ μl^-1^ of BSA, and 1 Unit of AmpliTaq DNA polymerase (Applied Biosystems, Foster City, CA, United States) for AOB 16S rRNA; and 10 μl of Failsafe Green Premix F (Epicentre, Madison, WI, United States), 1 μM of each primer, and 0.5 Units of AmpliTaq DNA polymerase (Applied Biosystems, Foster City, CA, United States) for *Nitrospira nxrB*. The amount of DNA used in each reaction ranged from 0.32 to 53.3 ng of DNA for all samples (average was 11.9 ng per reaction; two samples had <1 ng).

Standard curves for the 16S rRNA assay were generated using linearized plasmid DNA from a cloned Iron Springs AOB 16S rRNA gene sequence. Standards for the archaeal *amoA* and *Nitrospira nxrB* assays were generated by gene synthesis (Life Technologies Corporation) from *Nitrosoarchaeum limnia* SFB1 *amoA* and *Nitrospira defluvii nxrB* sequences. The standard gene copies for the assays ranged from 13 – 1.2 × 10^7^ for archaeal *amoA*, 16 – 3.3 × 10^7^ for AOB 16S rRNA, and 15 – 1.5 × 10^7^ for *Nitrospira nxrB*. Duplicate or triplicate reactions were carried out for all the samples and standards, and average values were calculated. Melt curves were generated after each SYBR assay to check the specificity of amplification. For the standard curves, PCR efficiencies averaged 79–91% and the correlation coefficients (*R*^2^) were >0.99. Samples that amplified but fell below the lowest standard (e.g., <13 copies for archaeal *amoA*) were identified as “below detection limit.” “Non-specific amplification” was identified in the melt curve analysis.

### Illumina MiSeq Library Preparation

DNA extracts were sent to the University of Illinois Roy J. Carver Biotechnology Center, Urbana, Illinois, United States for amplicon sequencing on the Illumina MiSeq sequencing platform. Library preparation was completed with the Fluidigm 48.48 Access Array IFC platform (Fluidigm Corporation, South San Francisco, CA, United States) to amplify the products from eight different PCR primer sets: Arch349F/Arch806R for archaeal 16S rRNA (Takai and Horikoshi, 2000), V4_515F/926R for total 16S rRNA (Parada et al., 2016), NitA/CTO654R for AOB 16S rRNA (Voytek and Ward, 1995; Kowalchuk et al., 1997; Sonthiphand et al., 2013), FGPS872F/FGPS1269R for *Nitrobacter* 16S rRNA (Degrange and Bardin, 1995), nxrB169F/nxrB638R for *Nitrospira nxrB* (Pester et al., 2014), and Arch_amoAF/Arch_amoAR for archaeal *amoA* (Francis et al., 2005). The sequencing center validated each primer set to ensure amplification under the given reaction conditions prior to sequencing.

Samples were diluted to a final concentration of 2 ng ∗ μL^-1^. A mastermix was prepared with Roche (Basel, Switzerland) High Fidelity Fast Start Kit and 20x Access Array loading reagent according to Fluidigm protocols. Into each well of a PCR plate, 1 μL of each sample was mixed with 1 μL of Fluidigm Illumina linkers and unique barcodes mix, 0.5 μL of 10X FastStart Reaction Buffer without MgCl_2_, 0.9 μL of 25 mM MgCl_2_, 0.25 μL of DMSO, 0.1 μL of 10 mM PCR grade nucleotide mix, 0.05 μL of 5 U ∗ μL^-1^ FastStart High Fidelity enzyme blend, 0.25 μL of 20X Access Array Loading Reagent, and 0.95 μL of water. In a separate plate, 20X primer solutions were prepared by adding 2 μL of each forward and reverse primer (synthesized by IDT Corp, Coralville, IA, United States), 5 μL of 20X Access Array Loading Reagent, and 91 μL of water.

Once the sample mixture was complete, 4 μL was loaded into the sample inlets and 4 μL of the primer solution was loaded into the primer inlets of a primed Fluidigm 48.48 Access Array IFC. The IFC was then placed in a Fluidigm AX controller for microfluidic mixing of each primer and sample combination before being loaded into the Fluidigm Biomark HD PCR machine. Amplicons were generated using the following Access Array cycling program without imaging: 50°C for 2 min, 70°C for 20 min, 95°C for 10 min; 10 cycles of 95°C for 15 s, 60°C for 30 s, and 72°C for 1 min; 2 cycles of 95°C for 15 s, 80°C for 30 s, 60°C for 30 s, and 72°C for 1 min; 8 cycles of 95°C for 15 s, 60°C for 30 s, and 72°C for 1 min; 2 cycles of 95°C for 15 s, 80°C for 30 s, 60°C for 30 s, and 72°C for 1 min; 8 cycles of 95°C for 15 s, 60°C for 30 s, and 72°C for 1 min; and 5 cycles of 95°C for 15 s, 80°C for 30 s, 60°C for 30 s, and 72°C for 1 min.

After amplification, 2 μL of Fluidigm Harvest Buffer was added to each sample inlet, and the IFC loaded onto the AX controller to harvest all PCR products from each sample (e.g., all primer amplifications pooled together for each sample). The PCR products were quantified using Qubit and stored at -20°C. The samples were run on a Fragment Analyzer (Advanced Analytics, Ames, IA, United States) to confirm the expected sizes of amplicons. All of the 48 samples (containing all primer amplifications pooled together) were then pooled together in equal DNA concentrations into one tube. The pooled product was size selected on a 2% E-gel (Life Technologies Corporation), then recovered based on expected fragment size with a Qiagen (Hilden, Germany) gel extraction kit. Cleaned, size-selected products were run on an Agilent Bioanalyzer to confirm the expected profile and determine the average product size.

The size-selected pool was qPCR quantitated and loaded onto one MiSeq flowcell using a MiSeq 600-cycle sequencing kit, version 3 for 300 bp paired-end sequencing using a MiSeq FGx system in RUO mode. After sequencing, read data was translated into FASTQ files using the Illumina bcl2fastq 1.8.4 software with an ASCII offset of 33. PhiX DNA reads (used as a spike-in control) were removed by alignment to the PhiX genome. The Roy J. Carver Biotechnology Center used in-house scripts for sorting the reads (with two mismatches allowed in the 5′ primer sequences) and demultiplexing (with one mismatch allowed in the index sequence attached in library prep).

### Sequence Controls

Sequence reads were evaluated for each gene set in a no-template control sample (nuclease free water). A total of 24 Bacterial 16S rRNA OTUs were found in the blank, all of which were related to *Pseudomonas*. One of these OTUs had 7,000 sequence reads (compared to >15,000 reads for all of the AMD samples), but all other OTUs had low read counts (2–186 reads per OTU). No Archaeal 16S rRNA, AOA *amoA*, or *Nitrospira nxrB* OTUs were present in the blank. One sample (Carib03Sept14) was sequenced twice to evaluate reproducibility. Bacterial 16S rRNA gene community membership and relative abundance were highly correlated (*R^*2*^* > 0.99) between the replicate samples.

### Community Composition Analyses

Relative sequence abundance and diversity analyses were conducted using QIIME (Quantitative Insights into Microbial Ecology) (Caporaso et al., 2010) and UPARSE (Edgar, 2013). The paired-ends were joined and quality filtered at Phred quality score of 20. Singletons were removed with the min_count_fraction option in QIIME. Sequences with minimum merge length <80 bp were discarded and the last 20 bp were removed from both ends. Analyses for archaeal *amoA* were done based on sequencing read one due to the lack of overlap between read one and read two (gene amplicon length: 635 bp). Sequencing reads were clustered into operational taxonomical units (OTUs) at 97% sequence identity. For 16S rRNA genes, the final OTUs were checked for chimeras using the DECIPHER web tool with the short sequences option (Wright et al., 2012) and putative chimeras were removed from the OTU table. Taxonomy of OTUs was assigned using a BLAST-based method with the Greengenes 13_8 rep set database (DeSantis et al., 2006) for 16S rRNA and NCBI database for functional genes. For further analyses, the final OTUs were filtered for specific taxa: the class *Thaumarchaeota* was filtered from the Archaeal 16S rRNA reads, the order *Nitrosomonadales* was filtered from the AOB 16S rRNA reads (no other AOB sequences were identified in the dataset), and Bacteria were filtered from the total 16S rRNA reads.

The number of observed OTUs for each gene was determined using QIIME. The sequence threshold cutoffs were set to 15,680 sequences for the combined V4-V5 region of bacterial 16S rRNA, 650 sequences for *Nitrosomonadales* taxa-specific AOB 16S rRNA, 60 sequences for archaeal 16S rRNA, 60 sequences for *Thaumarchaeota* taxa-specific archaeal 16S rRNA, 70 sequences for archaeal *amoA*, and 500 sequences for *Nitrospira nxrB* (low sequence thresholds were set in order to maximize OTU recovery). Rarefaction was performed at multiple sequence depths between one and the rarified depth (set to the median number of sequences for each gene). Chao1 richness estimates were determined at rarified depth for each gene.

### Phylogenetic Analyses

Representative sequences of the observed OTUs were aligned and sequences with short length, internal stop codons, or misalignments were removed. The *Nitrospira nxrB* OTUs were aligned to the curated alignment created by Pester et al. (2014) using ARB (Ludwig, 2004). The alignment length for each gene was 260 bp for archaeal *amoA*, 480 bp for AOB 16S rRNA, and 445 bp for *Nitrospira nxrB*. Maximum Likelihood trees were constructed for representative sequences of observed OTUs using FastTree v2.1.5 package (Price et al., 2010) in Geneious v8.1.8 (Kearse et al., 2012), with Jukes-Cantor Correction and 100 bootstrap replicates. Although significant bootstrap values (>50) occurred at the majority of the major branching points, values were not plotted on the phylogenetic trees due to the limitations of phylogenetic analyses with short gene sequences. Trees were visualized using the Interactive Tree of Life (iTOL) (Letunic and Bork, 2016). The normalized average relative abundance was plotted for each OTU (OTU relative abundance averaged by region, then divided by the sum of the averages for all regions).

### Statistical Analyses

Spearman correlation between environmental parameters and total gene abundances (based on qPCR) was performed using the IBM SPSS Statistics software package version 23 (IBM, Armonk, NY, United States) with critical *p*-values (α) corrected using Bonferroni correction (the adjusted significant α-value was set to ≤0.00156). Spearman correlations between environmental parameters and the relative abundance of individual OTUs were carried out using QIIME (observation_metadata_correlation.py) with *p*-values corrected using the Bonferroni correction. The environmental parameters from surface sediments included in the statistical analyses were: pH, temperature (°C), conductivity (μS ∗ cm^-1^) and dissolved oxygen (mg ∗ L^-1^). Environmental parameters from surface water included in statistical analyses were: TRW (in μg ∗ L^-1^) including Al, As, Ba, Cd, Ca, Cu, Fe, Pb, Mg, Mn, Ni, Na, Sr, Tl, and Zn; and DM (in μg ∗ L^-1^) including Al, Ba, Cd, Ca, Cu, Fe, Pb, Mg, Mn, Ni, Na, Sr and Zn.

Correlations between nitrifier community composition (AOA *amoA*, AOB 16S rRNA, and *Nitrospira nxrB* genes) and environmental parameters were analyzed using canonical correspondence analysis (CCA) with the Canoco 5 v. 5.04 (Šmilauer and Lepš, 2014). Relative abundance of OTUs was used as the species input. Environmental variables included in the analyses were pH, conductivity, temperature, dissolved oxygen, and TRW for Al, Ca, Fe, Pb, Mg, Mn, Na, Sr, Zn (dissolved metals were removed because of the covariation with total metals). Significant environmental variables were determined through forward selection with adjusted *p-*values (Bonferroni Correction).

### Nucleotide Sequence Accession Numbers

Sequences reported in this study have been deposited in GenBank under accession numbers KY938713-KY938805; KY940445-KY940462; and KY942196-KY944567.

## Results

### Chemistry

Sample sites covered four different mines/regions within the greater Iron Springs Mining District: Caribbeau Mine (Carib01, Carib02 and Carib03), Iron Bog/Fen (FenIB01, FenIB02, HFIBE and Opp03), New Dominion Mine (FenND01, FenND03, NDMD02, NDCS02, CS1B and NDGP), and Howard Fork River (HF04) (**Figure 1**). The measured environmental parameters varied by sampling date and sampling location (Supplemental Tables S1, S2). Across surface sediments from all the sites in 2013 and 2014, temperature ranged from 7.1°C – 21.2°C, dissolved oxygen ranged from 1.1 – 10 mg ∗ L^-1^, and pH ranged from 3.2 – 8.3. The lowest pH was measured at the CS1B site in June of 2013 and 2014 (pH 3.2). The Caribbeau Mine sites had circumneutral pH at all time points (pH 7.3 on average).

Metal concentrations from surface water (TRW and DM) were measured for all samples sites and time points (Supplementary Tables S1, S2). The sediments contained high concentrations of metals commonly found in AMD environments including cadmium, copper, iron, lead, and zinc. Overall, New Dominion Mine sites had the highest average metal concentrations for 100% of the metals in 2013 and 80% of the metals in 2014, including Ca, Fe, Mg, Mn, Na, Sr, and Zn (for metals measured in at least 80% of the samples).

### Gene Abundance of AOA, AOB, and NOB

**FIGURE 2:**
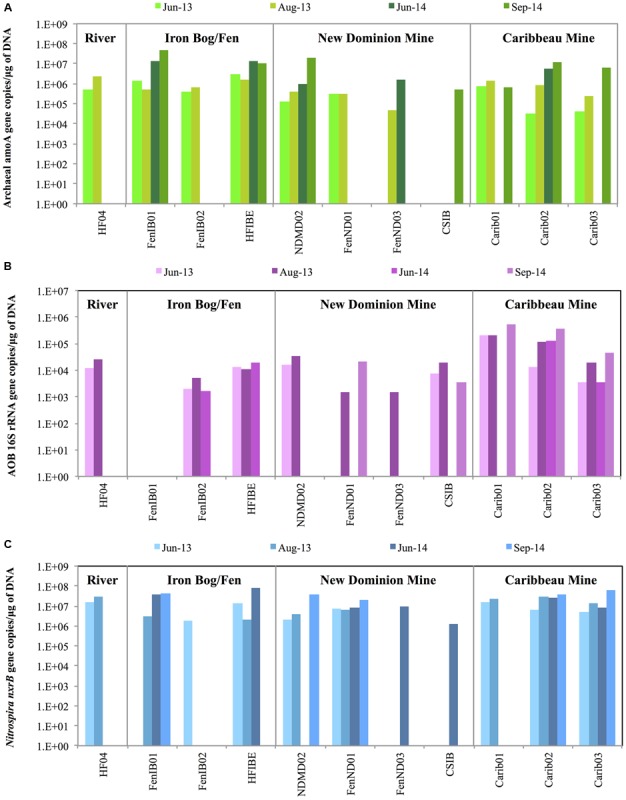
The overall abundance of **(A)** archaeal *amoA* genes, **(B)** AOB 16S rRNA genes, and **(C)** *Nitrospira nxrB* genes across 2013 and 2014 samples sites in the Iron Springs Mining District, based on quantitative PCR. Values represent averages of duplicate or triplicate qPCR reactions. The *y*-axis is represented in logarithmic scale.

The overall abundance of AOA, AOB, and NOB genes was determined across a total of 38 AMD-impacted sites from June 2013, August 2013, June 2014, and September 2014 from the Iron Springs Mining District (**Figure 2** and Supplementary Table S3). Gene abundance showed a clear seasonal pattern: AOA, AOB, and NOB genes were more abundant in late summer than in early summer (∼5 times more abundant in late summer for ∼80% of samples where abundance was measured in both time points for a given year).

Archaeal *amoA* gene copies across 2013 and 2014 ranged from 3.2 × 10^4^ – 4.9 × 10^7^ copies ∗ μg DNA^-1^ (**Figure 2A**). Archaeal *amoA* genes were most abundant in the Iron Bog/Fen sites (HFIBE and FenIB01) during both 2013 and 2014, ranging from 5.4 × 10^5^ – 4.9 × 10^7^ copies ∗ μg DNA^-1^. Archaeal *amoA* gene copy numbers were lowest at the New Dominion Mine FenND03 site in 2013 with 4.8 × 10^4^ copies ∗ μg DNA^-1^, with a ∼1,000-fold difference when compared to the most abundant sites.

Ammonia-oxidizing bacteria were quantified using AOB-specific 16S rRNA gene primers since AOB *amoA* genes did not amplify in our samples [using primers amoA-1F/amoA–2R (Rotthauwe et al., 1997) or amoA–1F∗/amoA–2R (Stephen et al., 1999)]. The overall abundance of AOB 16S rRNA genes across 2013 and 2014 samples sites ranged from 1.5 × 10^3^ – 5.3 × 10^5^ copies ∗ μg DNA^-1^ (**Figure 2B**). During 2013 and 2014, the AOB 16S rRNA genes were most abundant in the Caribbeau Mine sites, ranging from 3.5 × 10^3^ – 5.3 × 10^5^ copies/μg DNA. The AOB 16S rRNA gene copy numbers were lowest at the New Dominion Mine sites (FenND01 and FenND03, 2013) with 1.5 × 10^3^ copies ∗ μg DNA^-1^.

The qPCR results indicated that archaeal *amoA* genes were more abundant than AOB 16S rRNA genes at most sites and time points, which may suggest that AOA are more abundant than AOB since both genes are thought to exist in single copies within each genome (Aakra et al., 1999; Kowalchuk and Stephen, 2001; Hermansson and Lindgren, 2001; Hallam et al., 2006; Norton et al., 2008). Archaeal *amoA* genes were more abundant than AOB 16S rRNA genes in 100% of the samples where both genes amplified (*n* = 22; 100 times more abundant on average).

*Nitrospira nxrB* gene copies ranged from 1.3 × 10^6^ – 7.7 × 10^7^ copies ∗ μg DNA^-1^ across 2013 and 2014 (**Figure 2C**). During 2013, *Nitrospira nxrB* genes were most abundant at the Howard Fork River sites ranging from 1.6 × 10^7^ – 2.9 × 10^7^ copies ∗ μg DNA^-1^ and Caribbeau Mine sites ranging from 5.3 × 10^6^ – 2.8 × 10^7^ copies ∗ μg DNA^-1^. In 2014, *Nitrospira nxrB* genes were most abundant at the Iron Bog/Fen sites (FenIB01 and HFIBE) ranging from 3.8 × 10^7^ – 7.7 × 10^7^ copies ∗ μg DNA^-1^ followed by Caribbeau Mine sites (Carib02 and Carib03) ranging from 8.8 × 10^6^ – 6.5 × 10^7^ copies ∗ μg DNA^-1^. *Nitrospira nxrB* gene copy numbers were the lowest at New Dominion Mine sites (1.3 × 10^6^ – 3.8 × 10^7^ copies ∗ μg DNA^-1^) when compared across all sample regions during 2013 and 2014.

*Nitrobacter* genes did not amplify in any of the samples using nxrB-1F/nxrB-1R primers (Vanparys et al., 2007), *Nitrobacter*–specific 16S rRNA gene primers Nitro-1198f/Nitro-1423r (Huang et al., 2010), or *Nitrobacter*–specific 16S rRNA gene primers FGPS872f/FGPS1269r (Degrange and Bardin, 1995).

### Relative Abundance, Richness, and Phylogeny

Sequence analyses revealed that bacterial 16S rRNA genes were related to *Proteobacteria* (47%), *Bacteroidetes* (10%), *Chloroflexi* (9%), *Acidobacteria* (8%), and *Actinobacteria* (7%). *Nitrospira* accounted for 0.4% and *Nitrosomonadaceae* accounted for <0.1% of the total bacterial 16S rRNA community. No *Nitrobacter* sequences were found in the bacterial 16S rRNA gene dataset. A total of 2286 unique bacterial 16S rRNA gene OTUs were identified (at 97% sequence identity), with 181-1291 OTUs observed within each sample (Supplementary Figures S2A, S3A). Chao1 richness estimates ranged from 209 – 1384 OTUs for each sample.

The archaeal 16S rRNA gene sequences were related to the phyla *Crenarchaeota* (58%) including the *Thaumarchaeota* (42%), *Euryarchaeota* (16%), and *Parvarchaeota* (26%). A total of 66 unique archaeal 16S rRNA gene OTUs were identified, with 1–22 OTUs observed within each sample (Supplementary Figures S2B, S3B). Chao1 richness estimates ranged from 1 – 20 OTUs for each sample.

**FIGURE 3:**
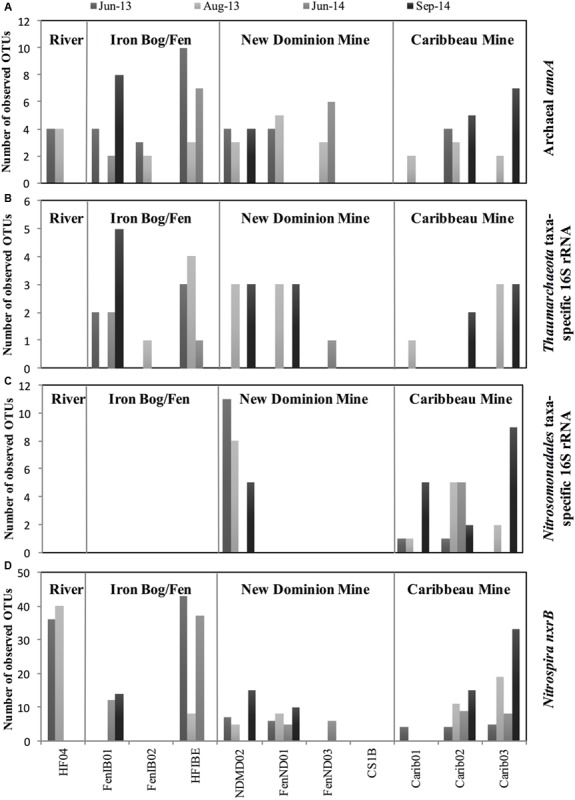
Number of observed OTUs at each site across 2013 and 2014 for **(A)** Archaeal *amoA*, **(B)** *Thaumarchaeota* taxa-specific 16S rRNA, **(C)** *Nitrosomonadales* taxa-specific 16S rRNA, and **(D)** *Nitrospira nxrB* genes. Samples not sequenced were: HF04-June14; HF04-Sept14; FenND03-Jun13; FenND03-Sept14; FenIB02-Sept14; Carib01-June14. All other samples with zero values did not amplify.

**FIGURE 4:**
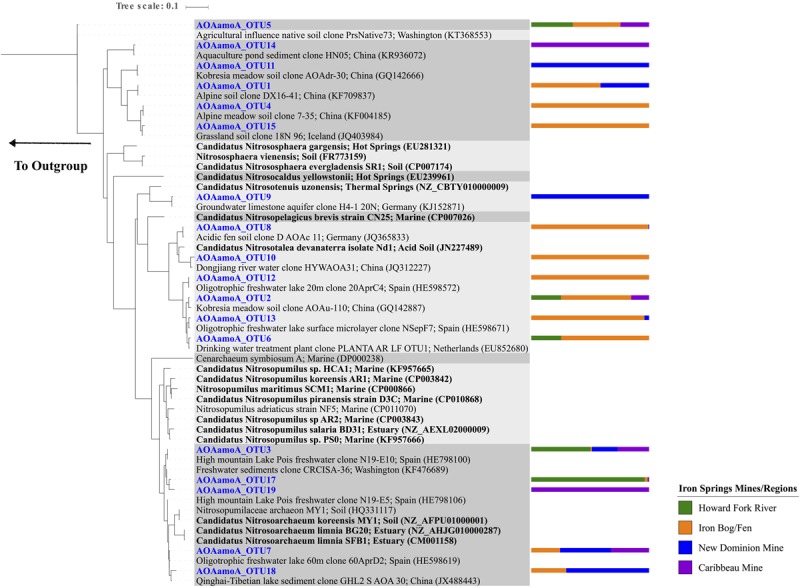
Maximum Likelihood phylogenetic tree (based on a 260 bp nucleotide sequence alignment) showing the affiliation of Iron Springs archaeal *amoA* gene sequences (in blue) and other environmental and culture sequences (cultured organisms in bold). NCBI accession numbers are denoted in parentheses. The bar graph represents the normalized relative abundance across sample regions per each OTU. The outgroup leads to *Nitrosomonas cryotolerans* (AF314753).

For archaeal *amoA* genes, 18 OTUs were recovered across all samples, with 2–10 OTUs observed within each sample (**Figure 3A** and Supplementary Figure S3C). Chao1 richness estimates ranged from 1 – 9 OTUs for each sample at their highest sequencing depths. The 18 observed OTUs were phylogenetically related to *Nitrosoarchaeum* (5 OTUs), *Nitrosotenuis* (1 OTU), *Nitrosotalea* (6 OTUs), and soil clones that had 82.7% sequence identity to *Nitrososphaeara* (6 OTUs) (**Figure 4** and Supplementary Table S4). Five OTUs (OTU 3, 2, 5, 17, and 19) were present at all four sampling site regions. Six OTUs were observed only in one region (OTU 10, 12, and 15 found only at the Iron Bog/Fen sites; OTU 14 found only at the Caribbeau Mine site; and OTU 9 and 11 found only at the New Dominion Mine sites). The remaining 7 OTUs were found in two or three sampling regions. For *Thaumarchaeota* 16S rRNA genes (filtered from the total Archaeal 16S rRNA gene set), 8 OTUs were recovered across all samples, with 1–5 OTUs observed within each sample (**Figure 3B**, Supplementary Figure S3D and Table S5). Chao1 richness estimates ranged from 1 – 5 OTUs for each sample at their highest sequencing depths. The observed *Thaumarchaeota* 16S rRNA gene OTUs had highest nucleotide identity to uncultured archaeal clones (based on BLAST), and ranged from 80 to 94% nucleotide identity to *Nitrosopumilus maritimus*—the first cultured AOA.

**FIGURE 5:**
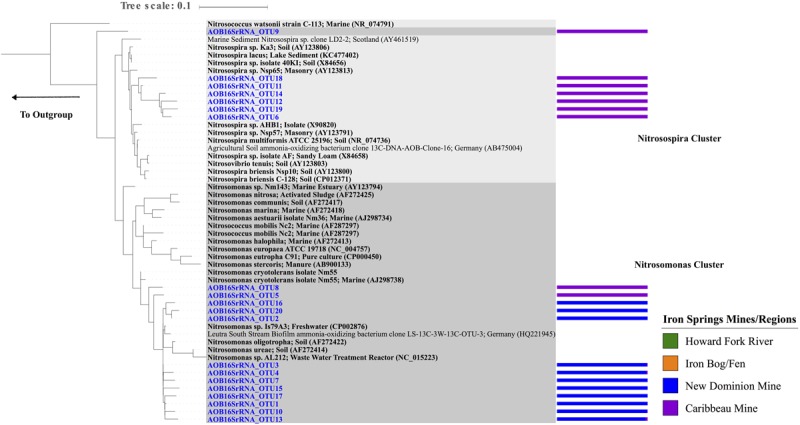
Maximum Likelihood phylogenetic tree (based on a 480 bp nucleotide sequence alignment) showing the affiliation of Iron Springs *Nitrosomonadales* taxa specific 16S rRNA gene sequences (in blue) and other environmental and culture sequences (cultured organisms in bold). NCBI accession numbers are denoted in parentheses. The bar graph represents the normalized relative abundance across sample regions per each OTU. The outgroup leads to *Bradyrhizobium japonicum* NA6086 (AB072418).

A total of 20 unique AOB 16S rRNA gene OTUs were recovered belonging to the order *Nitrosomonadales*, with 1–11 OTUs observed within each sample (**Figure 3C** and Supplementary Figure S3E). Chao1 richness estimates ranged from 1 – 10 OTUs for each sample. These OTUs were only found in two regions: New Dominion Mine and Caribbeau Mine. In all cases, relative abundance within an OTU was far greater at one region over the other (>97% normalized relative abundance), with 11 OTUs dominating at New Dominion Mine and 9 OTUs dominating at Caribbeau Mine. Phylogenetic analyses indicated that the New Dominion Mine OTUs were only associated with *Nitrosomonas* (**Figure 5** and Supplementary Table S6). Several OTUs were closely related to a cluster of sequences found in low nutrient environments. Caribbeau Mine OTUs were related to *Nitrosospira* and *Nitrosomonas* (**Figure 5**).

**FIGURE 6:**
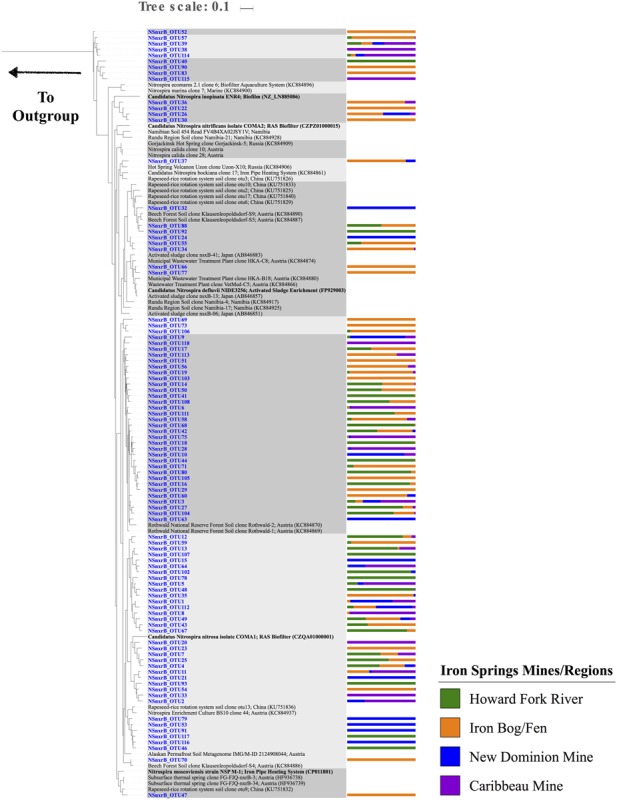
Maximum Likelihood phylogenetic tree (based on a 445 bp nucleotide sequence alignment) showing the affiliation of Iron Springs *Nitrospira nxrB* gene sequences (in blue) and other environmental and culture sequences (cultured organisms in bold). NCBI accession numbers are denoted in parentheses. The bar graph represents the normalized relative abundance across sample regions per each OTU. The outgroup leads to *Candidatus Kuenenia stuttgartiensis* (CT573072).

*Nitrospira nxrB* sequences had the highest number of observed OTUs (compared to AOA and AOB), with a total of 93 unique OTUs and 4-43 OTUs found at each individual site (**Figure 3D** and Supplementary Figure S3F). Chao1 richness estimates ranged from 4 – 43 OTUs for each sample. Overall, there were 13 OTUs that were found in all four sampling regions. Thirty-three OTUs were only found at one sampling region. A majority of the OTUs were closely related to sequences from Rothwald National Reserve forest soil (Pester et al., 2014) (**Figure 6** and Supplementary Table S7). Six OTUs were closely related to sequences from an enrichment culture initiated from a wastewater treatment plant (BS10, KC884937) (Pester et al., 2014). Two OTUs clustered with *Nitrospira defluvii*, enriched from activated sludge. Several *nxrB* OTUs clustered with ‘*Candidatus* Nitrospira inopinata’ (89.3% pairwise sequence identity) and ‘*Candidatus* Nitrospira nitrosa’ (91.6% pairwise sequence identity), organisms recently implicated in the complete nitrification of ammonia to nitrite (comammox) (Daims et al., 2015; van Kessel et al., 2015). However, the exact phylogenetic placement of these OTUs varied slightly based on the maximum likelihood algorithm used. Nine OTUs clustered independently on the phylogenetic tree, and had low sequence identity (<85%) to *nxrB* database sequences.

*Nitrobacter* 16S rRNA gene sequence analyses revealed that one OTU was closely related (99% sequence identity) to sequences from a municipal wastewater *Nitrobacter* clone. One *Nitrobacter* OTU was also present in another Iron Springs sample used as technical sequencing replicate, as well as other OTUs with low read counts (2–69 per OTU). *Nitrobacter* sequences were not further analyzed due to the low number of sequences and samples with hits.

### Correlation with Environmental Parameters

For each nitrification gene dataset (AOA *amoA*, AOB 16S rRNA, *Nitrospira nxrB*), we evaluated the relationship between environmental parameters and the total gene abundances (qPCR), relative gene abundance for individual OTUs (based on gene sequence counts), and overall community composition (CCA of relative gene abundance). All correlation analyses used corrected *p-values* to account for the large number of comparisons.

Though pH levels varied significantly across all Iron Springs sites (ranging from 3.2–8.3), pH was not strongly correlated to the overall community structure or relative abundance of individual OTUs for any gene (based on CCA and Spearman correlation). pH was positivity correlated to the total abundance (qPCR) of AOB 16S rRNA genes, but not for any other genes (Supplementary Table S8).

The high concentrations of heavy metals in the Iron Springs sediments were not correlated with the overall nitrifier community composition or abundance (based on CCA and Spearman correlations). Nonetheless, the relative abundances of several individual *Nitrospira nxrB* and AOB 16S rRNA OTUs, as well as one archaeal *amoA* OTU, were negatively correlated with metals including Mn, Zn, Sr and Pb (Supplementary Table S9). At the same time, the relative abundance of other *Nitrospira nxrB*, archaeal *amoA*, and AOB 16S rRNA OTUs was positively correlated with Mn and Cu (Supplementary Table S9).

Temperature, which ranged from 7.1–21.2°C across the sites, was a strong predictor of the AOB 16S rRNA community structure. CCA showed that the overall composition of the AOB 16S rRNA community was strongly correlated with temperature (Supplementary Figure S4). Total AOB 16S rRNA gene abundances (qPCR) were negatively correlated with temperature (Supplementary Table S8). Temperature was positively correlated to the relative abundance of three individual AOB 16S rRNA OTUs (OTU 3, 7, and 16). Temperature was negatively correlated to the relative abundance of one *Nitrospira nxrB* OTUs (OTU 28) (Supplementary Table S9).

The *Nitrospira nxrB* community structure was correlated with conductivity or conductivity-related ions (e.g., Na, Ca, Mg) in several ways. CCA showed that the overall composition of the AOB 16S rRNA and *Nitrospira nxrB* communities was strongly correlated with conductivity (Supplementary Figure S4). The relative abundance of several individual *Nitrospira nxrB* was negatively correlated with conductivity or conductivity-related ions, and one *Nitrospira nxrB* OTU (OTU 1) was positively correlated with Ca (Supplementary Table S9). Sodium was also correlated with the overall composition of the AOB 16S rRNA genes (CCA, Supplementary Figure S4).

## Discussion

Acid mine drainage is a serious threat to freshwater systems and can devastate a river and its aquatic life through its acidic pH and high metal concentrations. In this study, we determined the abundance and diversity of AOA, AOB, and NOB gene sequences through DNA-based analyses in AMD-impacted sediments at the Iron Springs Mining District. The abundance and diversity of AOA, AOB, and NOB genes in these AMD-impacted sediments may suggest the potential for nitrification activity.

Low pH presents physiological challenges to nitrifying microbes through reduced bioavailability of ammonia and nitrite (Suzuki et al., 1974; Stein et al., 1997). Nitrification is thought to be energetically challenging at low pH because ammonia (NH_3_) and nitrite (NO_2_^-^) concentrations are very low due to the chemical equilibrium in solution (NH_3_ + H^+^ → NH_4_^+^, pKa ∼9.24; NO_2_^-^ + H^+^ → HNO_2_, pKa ∼3.39). At low pH, the number of protons in solution increases and the equilibrium is shifted away from the energetic substrate (ammonia and nitrite). Here, AOA *amoA* and *Nitrospira nxrB* genes were present at most sites and time points regardless of pH. Archaeal *amoA* and *Nitrospira nxrB* gene abundances were high (10^3^ – 10^7^ copies per μg DNA) at sites with pH ≤ 4.5, and genes were detected at sites with pH as low as 3.5. Because pH reduces the bioavailability of ammonia, acidic habitats may favor AOA dominance over AOB due to their high affinity for ammonia (Martens-Habbena et al., 2009; Offre et al., 2014). Alternatively, it was recently proposed that some AOA might possess ammonium transporters instead of ammonia transporters found in AOB (Offre et al., 2014; Lehtovirta-Morley et al., 2016). Similarly, substrate availability and enzyme activity for nitrite oxidation are reduced as the pH decreases (Tanaka et al., 1983) often leading to greater NOB abundance at higher pH conditions. AOA and NOB could have additional mechanisms of adaptation to low pH, such as increased ureolytic activity, proton efflux proteins, proton consuming metabolisms, and cytoplasmic proteins with buffering capacity. The mixotrophic lifestyle of some nitrifiers (e.g., litho- and organotrophy; Hatzenpichler, 2012) may afford an additional means of overcoming energetic stress under low pH conditions through the utilization of alternate electron sources.

Metal concentrations (e.g., Al, Cu, Fe, and Zn) at all of the sites exceeded the allowable concentrations as determined by the Colorado Department of Public Health and Environment (CDPHE) Regulation 31. Sites with pH <4 had the highest concentrations of metals, perhaps because low pH can increase metal dissolution. Heavy metals are known to inhibit nitrification in some systems (Cela and Sumner, 2002; Yan et al., 2013); yet, we found high numbers of AOA, AOB, and NOB genes at sites with high metal concentrations. The relative abundance of some individual OTUs was negatively correlated with Mn, Zn, Sr and Pb, while other OTUs were positively correlated with Mn. One archaeal *amoA* OTU was positively correlated with copper, which is heavily involved in ammonia oxidation and electron transport for AOA (Walker et al., 2010). Specific metals did not equally impact gene abundances of AOA, AOB, or NOB, possibly suggesting that metals have different effects on each of these groups as seen in previous studies (Li et al., 2009; Subrahmanyam et al., 2014; Ouyang et al., 2016). For instance, Mn was negatively correlated with some AOB OTUs, positively correlated with some NOB OTUs, and showed no correlation with AOA OTUs. Other previous studies showed decreased abundance or changes in community composition with increasing metal concentrations (Mertens et al., 2006; Principi et al., 2008; Li et al., 2009; Cao et al., 2011). Though metal resistance genes have been found in some nitrifiers (Stein et al., 2007; Starkenburg et al., 2008; Lücker et al., 2010; Spang et al., 2012), it is currently unknown whether these nitrifiers have specific adaptations to tolerate high metal concentrations (e.g., siderophore production, extracellular sequestration, ATP-dependent efflux systems).

Nitrifier gene abundance showed interesting patterns in the Iron Springs mining region. Gene abundance showed a clear seasonal pattern: AOA, AOB, and NOB genes were more abundant in late summer than in early summer (∼5 times more abundant in late summer for ∼80% of samples where abundance was measured in both time points for a given year). Overall, AOA were more abundant than AOB across most sample regions irrespective of varying environmental conditions, which may be due to differences in AOA and AOB physiology. *Nitrospira* nitrite oxidizers were also abundant across all sample regions. When ammonia oxidizers and nitrite oxidizers were compared together, we observed that the *Nitrospira nxrB* genes were more abundant than archaeal *amoA* and AOB 16S rRNA genes at many sites (**Figure 2**). However, gene copy numbers vary for each organism: AOA have one *amoA* copy per cell, AOB have one 16S rRNA copy per cell, and *Nitrospira* have 2-6 *nxrB* copies per cell (Klappenbach et al., 2001; Lücker et al., 2010; Hatzenpichler, 2012; Pester et al., 2014). At a number of sites, we observed either no amplification or non-specific amplification (for archaeal *amoA*, AOB 16S rRNA, and *Nitrospira nxrB*), possibly suggesting that AOA, AOB and NOB were absent in those selected sites, present in low numbers, or did not properly amplify with the PCR primers. Further analyses would be necessary to confirm low nitrifier abundance and its ecological implications (e.g., accumulation of ammonia, decreased denitrification, changes in N_2_O emissions).

The overall bacterial 16S rRNA gene community structure in Iron Springs sediments was similar to microbial communities observed in other AMD environments (Volant et al., 2014; Méndez-García et al., 2015), but very little is known about the community structure of nitrifying microbes in AMD environments. In this study, the richness (numbers of observed OTUs) and phylogenetic diversity of the AOA, AOB, and NOB communities was comparable to other more moderate environments (e.g., uncontaminated soils or freshwater systems). Regions with acidic pH had several AOA OTUs related to *Nitrosotalea* associated with acidic soil environments (**Figure 4**) (Lehtovirta-Morley et al., 2011; Gubry-Rangin et al., 2011; Pester et al., 2012), which may suggest that these AOA are adapted to acidic pH conditions. Many of the OTUs found in the Howard Fork River and Caribbeau Mine sites (more neutral pH) were associated with freshwater sequences (van der Wielen et al., 2009; Auguet et al., 2012; Mosier et al., 2012; Auguet and Casamayor, 2013).

Ammonia-oxidizing bacteria observed in these AMD-impacted sites belonged to the family *Nitrosomonadaceae*, which are generally well distributed in both terrestrial and aquatic environments (Stein et al., 2007; Norton et al., 2008; Zheng et al., 2013) (**Figure 5**). The majority of OTUs dominating the Caribbeau Mine region clustered with *Nitrosospira* found in soil environments (Utåker et al., 1995; Jiang and Bakken, 1999; Purkhold et al., 2003; Norton et al., 2008). On the other hand, OTUs dominating the New Dominion Mine region belonged only to the *Nitrosomonas* cluster and were closely related to *Nitrosomonas sp.* Is79A3 usually found at sites with low ammonia concentrations (Bollmann et al., 2013). These findings could suggest that the AOB in the New Dominion Mine may be adapted to low substrate availability (ammonia), possibly a result of the low pH compared to the Caribbeau Mine.

NOB in these AMD-impacted sites were related to phylogenetically diverse *Nitrospira* species. *Nitrobacter* NOB genes did not amplify well in the AMD-impacted sediments (no qPCR amplification with several 16S rRNA and *nxrB* gene primer sets, and only a very small number of OTUs present in the sequence dataset). Although *Nitrobacter* are typically observed along with *Nitrospira* in aquatic systems, *Nitrospira* are often numerically dominant in sediments (Altmann et al., 2004; Cébron and Garnier, 2005; Freitag et al., 2006; Satoh et al., 2007) and water treatment plants (Juretschko et al., 1998; Daims et al., 2001; Huang et al., 2010). These findings may suggest that *Nitrobacter* are less tolerant of the harsh conditions in this system (low pH and high metals), that the available primer sets do not amplify the particular *Nitrobacter* found at these sites, or that biases in DNA extraction or PCR amplification prevented recovery of *Nitrobacter* sequences.

## Conclusion

In summary, we found interesting patterns of gene abundance and diversity of AOA, AOB, and NOB in AMD-impacted sediments with low pH and high metal concentrations. Sediment pH was correlated with the total abundance of AOB 16S rRNA genes, but not the total abundance of archaeal *amoA* or *Nitrospira nxrB* genes, or to the overall community structure or relative abundance of individual OTUs for any gene. Heavy metals, which had high concentrations in the Iron Springs sediments, were correlated with the relative abundances of several individual OTUs, but not the overall nitrifier community composition or abundance. It is important to note that many other factors not measured here could impact nitrification, including other chemical variables or organismal interactions. In addition, the combined effect of multiple, simultaneous stresses (e.g., acidic pH along with a suite of metals) on gene abundance and community composition is difficult to tease apart. This study provides a foundation for future work to determine rates of nitrification and the role of comammox bacteria in this AMD-impacted system, and to cultivate acidophilic and metal-tolerant AOA and NOB.

## Author Contributions

Conceived and designed the experiments: AM and TR. Sampling, site information, and chemistry: TR. Performed the experiments: BR. Analyzed the data: BR, AB, TR, and AM. Contributed to the manuscript: BR, AB, TR, and AM. Funded the research: AM and TR.

## Conflict of Interest Statement

The authors declare that the research was conducted in the absence of any commercial or financial relationships that could be construed as a potential conflict of interest.
