# Supplementary material for: Nitrifier Gene Abundance and Diversity in Sediments Impacted by Acid Mine Drainage
Source: Front Microbiol. 2017 Nov 7;8:2136. doi: 10.3389/fmicb.2017.02136 (PMC5701628; doi:10.3389/fmicb.2017.02136)
Supplement: Supplementary file 2 [file Image_1.PDF]

Supplementary Material

Nitrification Gene Abundance and Diversity in Sediments Impacted by Acid Mine Drainage

Bhargavi Ramanathan, Andrew M. Boddicker, Timberley M. Roane, and Annika C. Mosier

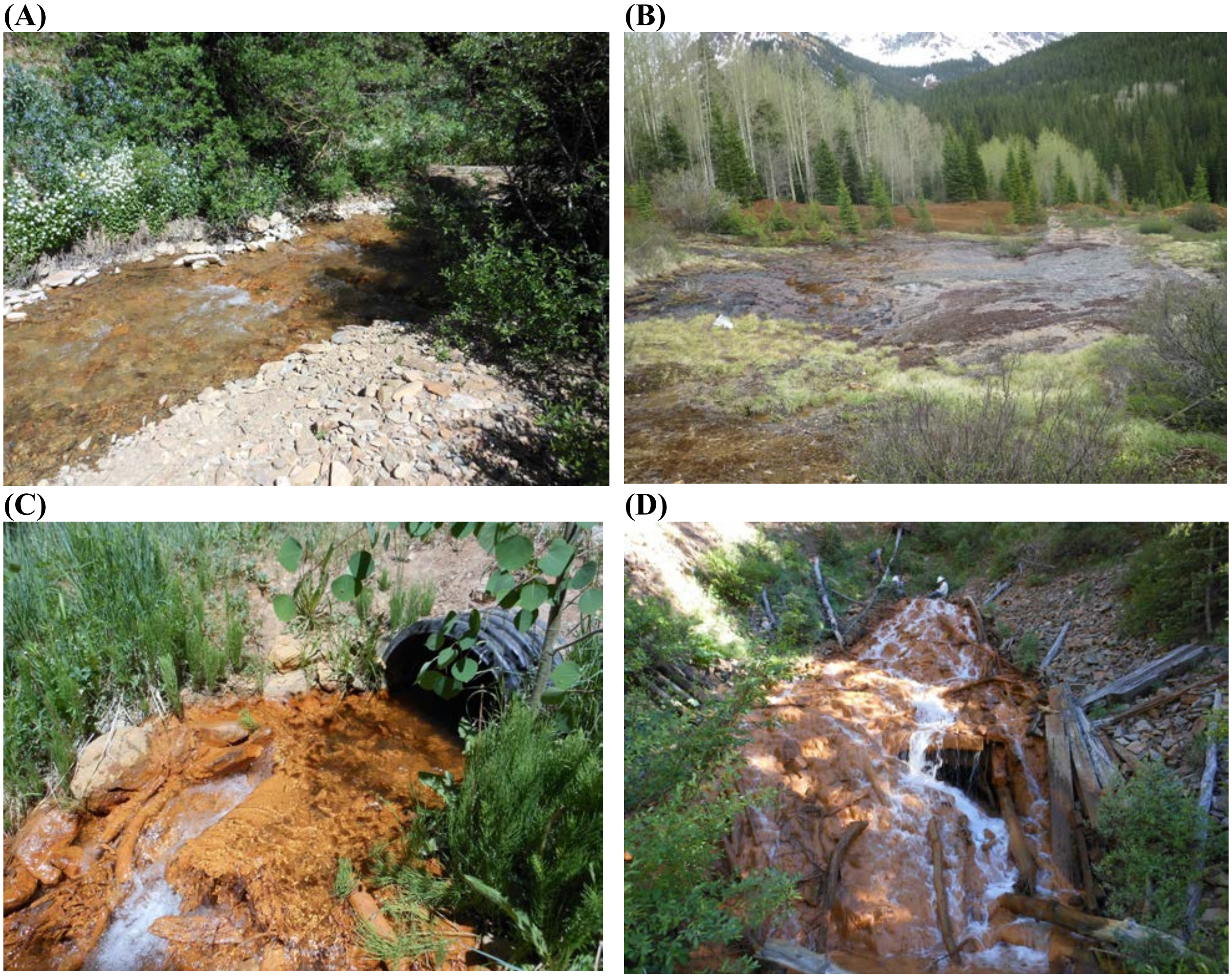

Supplemental Figure S1: Pictures of sample sites representative of each sample region (A) Howard Fork River, HF04; (B) Iron Bog/Fen, FenIB01; (C) New Dominion Mine, NDMD02; and (D) Caribbeau Mine, Carib01.

(A)

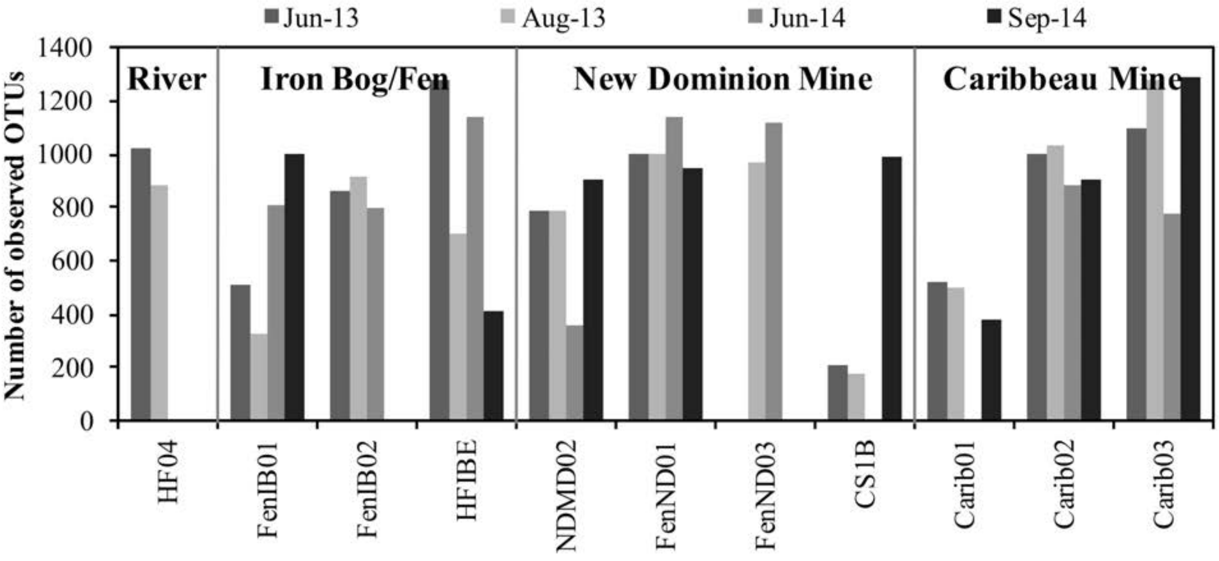

(B)

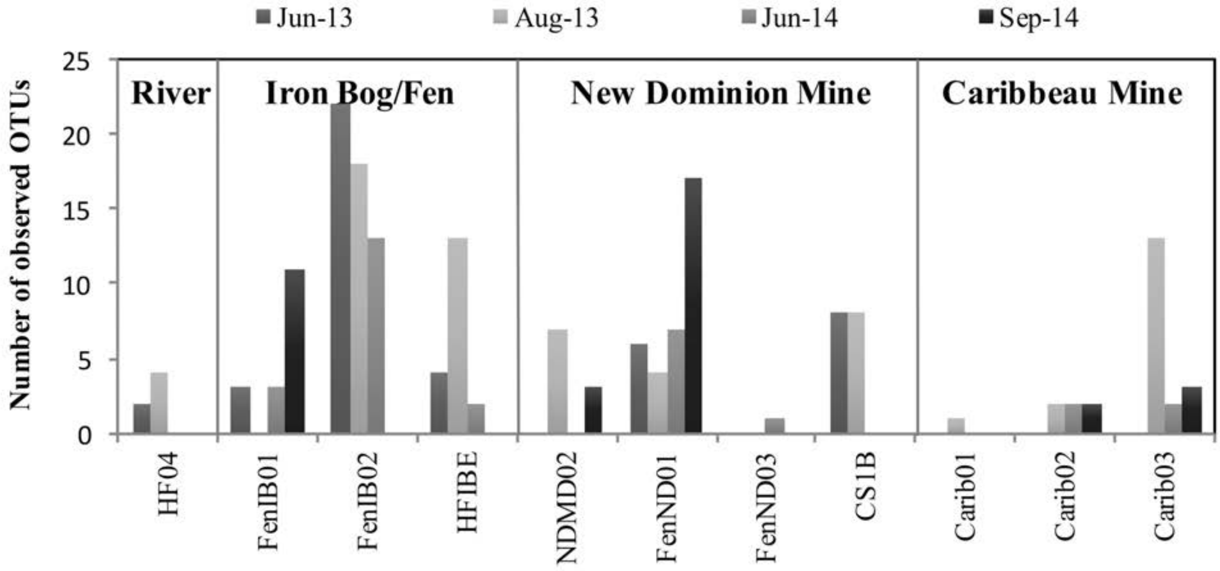

Supplemental Figure S2: Number of observed OTUs at each site across 2013 and 2014 for (A) Bacterial 16S rRNA, and (B) Archaeal 16S rRNA genes. Missing data points represent one of the following: the samples were not sequenced, the samples did not meet the sequence threshold set for each gene, or there were no OTUs detected in the samples.

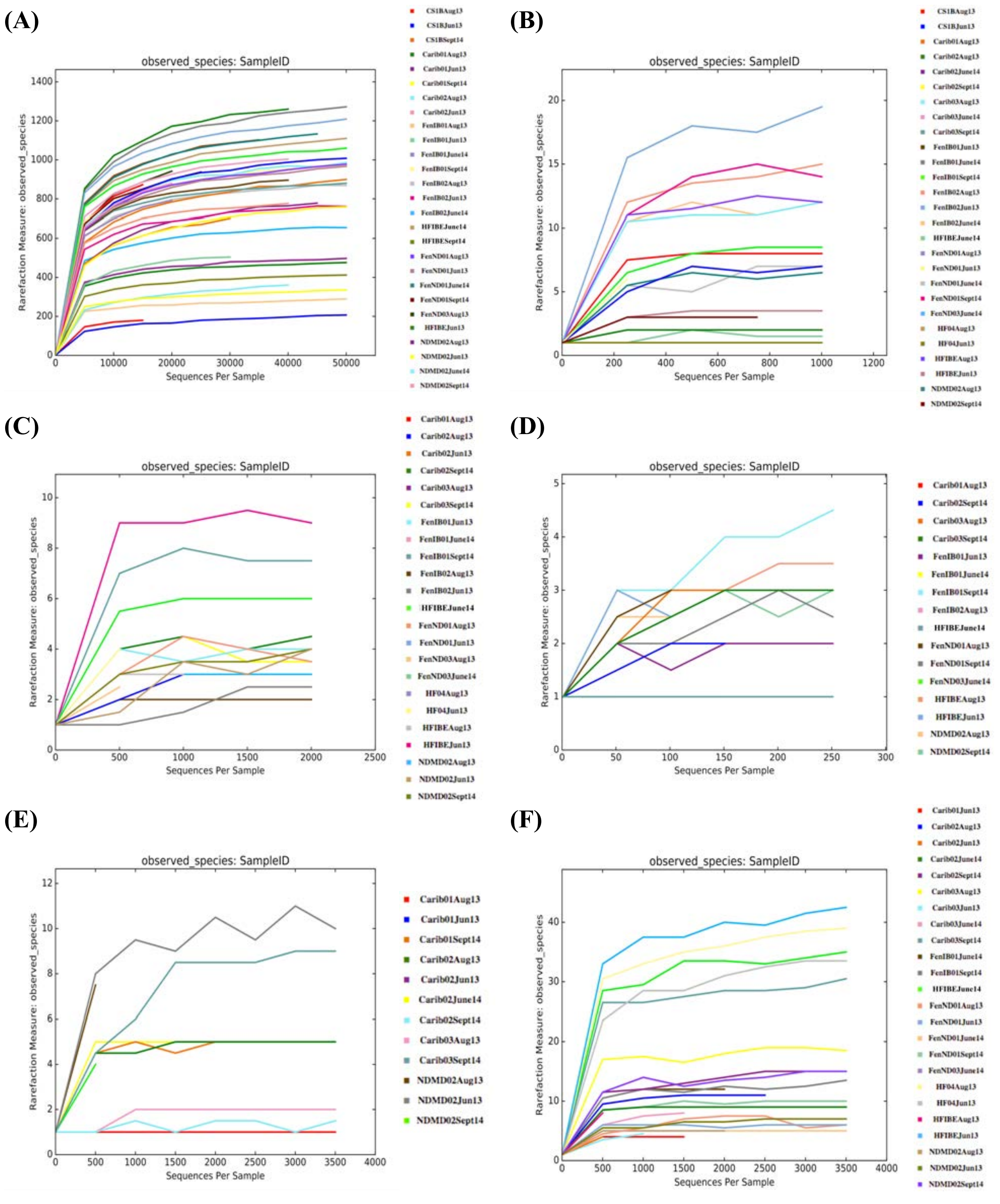

Supplemental Figure S3: Alpha rarefaction curves for each gene set: (A) bacterial 16S rRNA, (B) archaeal 16S rRNA, (C) archaeal *amoA*, (D) *Thaumarchaeota* taxa-specific 16S rRNA, (E) *Nitrosomondales* taxa-specific 16S rRNA, and (F) *Nitrospira nxrB*. The samples were rarefied between one and the median number of sequences for each gene.

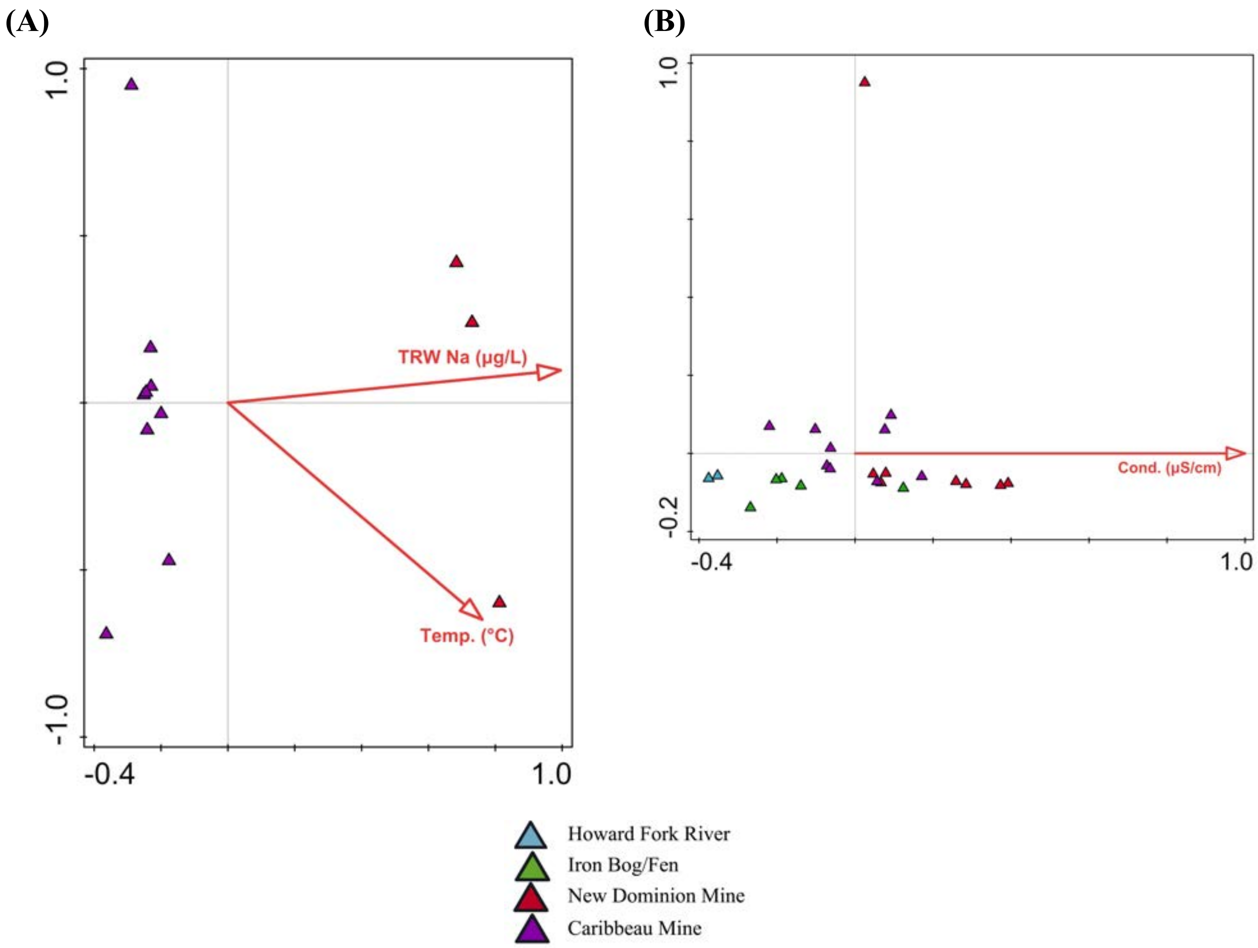

Supplemental Figure S4: Canonical Correspondence Analysis (CCA) of relative gene abundances and environmental variables: (A) *Nitrosomonadales* taxa-specific 16S rRNA gene during both years combined, and (B) *Nitrospira nxrB* gene during both years combined. Only analyses that showed significant Bonferroni-corrected *p*-values are included. 'TRW' represents the total recoverable metal concentrations.
